# First record of the human infection of *Brucella melitensis* in Kyrgyzstan: evidence from whole-genome sequencing-based analysis

**DOI:** 10.1186/s40249-022-01044-1

**Published:** 2022-12-01

**Authors:** Kalysbek Kydyshov, Nurbolot Usenbaev, Stalbek Berdiev, Aigul Dzhaparova, Aziza Abidova, Nuraiym Kebekbaeva, Murat Abdyraev, Gamal Wareth, Hanka Brangsch, Falk Melzer, Heinrich Neubauer, Mathias W. Pletz

**Affiliations:** 1 Institute of Bacterial Infections and Zoonoses, Friedrich-Loeffler Institute, Naumburger Str. 96a, 07743 Jena, Germany; 2 Republican Center for Quarantine and Highly Dangerous Infections of Ministry of Health, Bishkek, Kyrgyzstan; 3 Kyrgyz Scientific Research Institute of Veterinary Medicine, Bishkek, Kyrgyzstan; 4 Institute of Infectious Diseases and Infection Control, Jena University Hospital, Am Klinikum 1, Jena, Germany

**Keywords:** *Brucella melitensis*, Genome sequencing, Genotyping, Human, Kyrgyzstan

## Abstract

**Background:**

Brucellosis, a zoonosis mainly transmitted by consumption of unpasteurized dairy products as well as direct contact with infected animals, is endemic in Kyrgyzstan. However, *Brucella* species in humans have not been investigated and the origin of the disease remains poorly known in wide parts of Сentral Asia. Thus, molecular characterization of the circulating strains is a critical first step in understanding *Brucella* diversity in the country.

**Methods:**

In this study, isolates were collected from patients with suspected brucellosis from different regions in Kyrgyzstan between 2019 and 2020. The detection and identification of *Brucella* was carried out by Bruce-ladder PCR. Next generation sequencing was used to sequence the 89 *Brucella* isolates, which were genotyped by cgSNP and cgMLST to identify epidemiological connection between *Brucella* isolates as well as placing them in the context of the global *Brucella* phylogeny.

**Results:**

The *Brucella* strains isolated from all regions of Kyrgyzstan were identified as *B. melitensis.* Based on cgSNP analysis, 18 sequence types were differentiated. The highest numbers of different sequence types were found in Batken (*n* = 8), Osh (*n* = 8) and Jalal-Abad (*n* = 6) oblasts. According to cgSNP and cgMLST analyses, different *B. melitensis* lineages circulate in Kyrgyzstan, all of them belonging to the Eastern Mediterranean group of the global *Brucella* phylogeny with the highest similarity to strains from Turkmenistan, Iran and Turkey.

**Conclusion:**

In the present study, *B. melitensis* was identified as a causative agent of human brucellosis in Kyrgyzstan and different lineages could be identified. Since this study focused on isolates of human origin, the identity of *Brucella* species and lineages circulating among animal populations remains elusive. Implementing culture techniques and use of most recent molecular, bioinformatic and epidemiological tools are needed to set up a One Health approach to combat brucellosis in Kyrgyzstan. Further, other Сentral Asian countries need to take part in this effort as brucellosis is a transboundary disease in these regions.

**Graphical Abstract:**

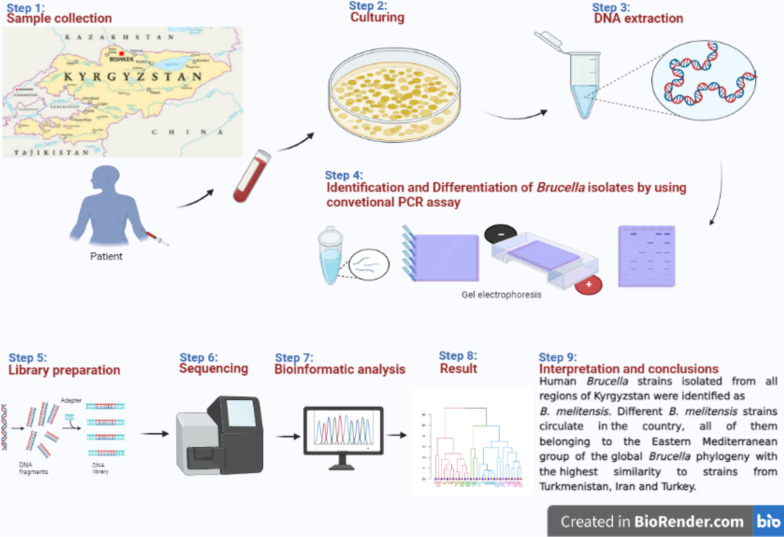

**Supplementary Information:**

The online version contains supplementary material available at 10.1186/s40249-022-01044-1.

## Background

Brucellosis causes prominent economic losses in animal production and serious disease in humans [1–3]. Currently twelve accepted *Brucella* species are recognized, and it has recently been suggested that *Ochrobactrum* spp., which are phylogenetically and taxonomically closest to *Brucella*, should be included in the *Brucella* genus as well. Species that cause the disease in humans are mainly *B. abortus* (reservoir in cattle), *B. melitensis* (reservoir in sheep and goats), *B. suis* biovars 1 and 3 (reservoir in pigs), and *B. canis* to some extent. Brucellae are Gram-negative, non-motile, and facultative intracellular coccobacilli that can infect a wide range of mammalian species including humans and some amphibians [4–7]. Humans are infected typically through contact with infected animals in rural areas, consumption of contaminated raw dairy products and inhalation of infected aerosolized particles in laboratories [8]. Brucellosis is often underreported, misdiagnosed and once a chronic form of the disease develops, it could be resistant to treatment, which results in long-term antibiotic administration. Mortality is reported to be low, but the disease can persist for several years and cause sequelae like arthritis [9, 10].

The number of human brucellosis cases worldwide exceeds 500,000 annually but the true incidence in some endemic countries can be expected to be higher due to unspecific clinical symptoms of the disease [11, 12]. Human brucellosis is a direct result of animal brucellosis and most cases are reported from countries of the Mediterranean Basin, the Middle East, Asia, Africa, and South America with hundreds or thousands of new cases every year [2, 8, 13–15].

Monitoring the prevalence of brucellosis in the human population is an important step toward identifying regions with high disease prevalence in animals and developing targeted measures for control and prevention. The traditional *Brucella* diagnostic system includes clinical examination, culturing of bacterial isolates from biological samples and serological tests such as Rose Bengal plate test (RBT), complement fixation test (CFT) and enzyme-linked immunosorbent assay (ELISA) [16–19]. Cultivation and bacterial phenotyping is considered the gold standard for diagnosis of brucellosis but is time consuming and pose a high risk to personnel involved in laboratory work [20]. A *B. abortus-melitensis-ovis-suis* polymerase chain reaction (AMOS-PCR) was the first species-specific PCR developed to differentiate *B. melitensis* (bv. 1, 2, and 3), *B. abortus* (bv. 1, 2, and 4), *B. ovis* and *B. suis* biovar 1 to reduce hands-on times [21]. However, methods for genetic characterization of bacterial pathogens have been developed using genome sequencing technologies, which allow a more detailed investigation of relationships between isolates. For example, next-generation sequencing (NGS) allows mass parallel sequencing, which was not possible with previous sequencing technologies [22–25]. Nowadays, whole-genome sequencing (WGS) and the determination of single nucleotide polymorphisms (SNPs) is a suitable tool for tracing *B. melitensis* infections [26]. *Brucella* spp. can be sub-typed by multilocus sequence typing (MLST) method, which targets a set of housekeeping genes. This method yields less detailed typing results, allowing only a rather global classification of the isolates. Therefore, core genome multilocus sequence typing (cgMLST) and core-genome SNP typing with higher discriminatory power has been developed to efficiently track the origin and spread of *Brucella* strains [27–32]. However, multiple-locus variable-number tandem repeat analysis (MLVA) is a widely used genotyping tool. The MLVA method is a non-sequence-based alternative for *Brucella* strain differentiation, and this method also allows phylogenetic analysis [33–37].

Kyrgyzstan, a former Soviet Republic, is located in Сentral Asia with a population of 6.5 million. More than 66% of Kyrgyzstan’s population is rural and 17% are employed in agriculture and livestock raising. Cattle, sheep, goats, horses, and poultry are the main livestock species and their products add a substantial contribution to the national economy of the country [38–40].

The Republican Center for Quarantine and Highly Dangerous Infections Ministry of Health (RCQHDI) is the authority responsible for human brucellosis control in Kyrgyzstan. Currently, serologic testing is the main surveillance method used for humans, i.e., the Huddleson plate agglutination test [41]. An investigation of the genetic relatedness, molecular epidemiology and protentional transmission route of brucellae from humans and animals has not yet been conducted in Kyrgyzstan. Thus, the purpose of this study was to investigate the genetic diversity of *Brucella* isolates using whole-genome sequencing for genotyping based on single nucleotide polymorphisms (SNPs) and core genome mulitlocus sequence typing (cgMLST) to determine epidemiological relationships between human *Brucella* isolates collected in Kyrgyzstan from December 2019 to November 2020. To the best of our knowledge, this is the first genotyping study conducted on human *Brucella* isolates from Kyrgyzstan.

## Methods

### Strain isolation and DNA extraction

Complete blood was taken from patients during the acute phase of brucellosis under aseptic conditions for haemocultivation as part of a standard clinical investigation at district or regional hospitals between 2019 and 2020. These samples were performed by Castaneda method [42] for sending to local oblast’s highly dangerous infections laboratories or branch of the bacteriology laboratories of the RCQHDI for culturing. During this period, a total of 198 positive cultures were sent to the RCQHDI reference laboratory for confirmation and further investigation (Additional file 5: Table S6).

Of these cultures, one hundred isolates were confirmed as a brucella positive culture. Further identification was done by colony morphology, aerobic or anaerobic growth, and by Gram and Stamp staining methods. Heat inactivated cultures were sent to the National and World Organisation for Animal Health (WOAH) Reference Laboratory for Brucellosis at the Institute of Bacterial Infections and Zoonoses of the Friedrich-Loeffler Institute Jena, Germany, for further investigation.

Genomic DNA was extracted from 200 µl heat-inactivated pure cultures using the High Pure PCR Template Preparation Kit (Roche, Mannheim, Germany) according to the manufacturer’s instructions. The genomic DNA concentration was quantified using a Qubit fluorometer (QubitTM DNA HS assay; Technologies Holdings Pte Ltd., Singapore). All isolates were analysed using a conventional *bscp31* PCR assay [43]. Bruce-ladder PCR was performed to identify the species of the isolates as described elsewhere [44].

### Generation and processing of sequencing data

Genomic libraries were prepared using the Nextera XT Library Preparation Kit (Illumina Inc., San Diego, CA, USA) and sequenced on a MiSeq system (Illumina Inc., San Diego, CA, USA) in paired-end mode. The resulting data were controlled for their quality using FASTQC v0.11.7 (https://www.bioinformatics.babraham.ac.uk/projects/fastqc/). De novo genome assembly was conducted using SPAdes within Shovill v1.0.4 (https://github.com/tseemann/shovill) and the assembly statistics were assessed by Quast v5.0.2 [45]. Using Prokka v1.14.5 potential open reading frames and RNA-coding genes were annotated in the newly generated genomes [46].

### Genotyping

In this study, cgSNP (core genome SNP) genotyping was used to investigate the molecular relationship between this study’s *B. melitensis* isolates and for placing these strains within the global *B. melitensis* phylogeny. Public databases, GenBank and NBCI were browsed for sequence data of *B. melitensis.* Data of geographically related (Asian) strains were downloaded and processed as described above. All in all, 65 foreign strains were included in the analysis (see Additional file 2: Table S2). The MLST sequence types of the Kyrgyz isolates were analyzed by 9-locus MLST using the software mlst (https://github.com/tseemann/mlst) [47] and a 16-loci multiple locus variable number of tandem repeats analysis (MLVA) as conducted using MISTReSS (https://github.com/Papos92/MISTReSS) [48]. For determining the position within the global *B. melitensis* phylogeny, a cgSNP (core genome SNP) analysis was conducted with *B. melitensis* 16 M (GCF_000007125.1) as a reference strain. Furthermore, a detailed cgSNP difference analysis was conducted by using the closely related strain BwIM_AFG_63 (GCF_002191235.1) as a reference. For these analyses, SNPs were called by Snippy v4.6.0 (https://github.com/tseemann/snippy) and maximum-likelihood trees were calculated using RAxML v8.2.12 [49].

Based on the SNP calling results, clusters of isolates were identified using hierClust (https://www.rdocumentation.org/packages/momr/versions/1.1/topics/hierClust). The trees were visualized and edited with iTOL v6.5.7 [50]. The Kyrgyz isolates were further compared to Asian *B. melitensis* strains by applying cgMLST using Ridom Seqsphere+ v7.7 [51] with the scheme of Janowicz et al. and default parameters [22]. Based on the allele profiles, a minimum spanning tree was created with pairwise ignoring missing values.

### Geographic map

The geographic maps were created using QGIS v3.22.5 Białowieża ( https://qgis.org/en/site/forusers/download.html, accessed on 18 March 2022), generated from GPS data in Google Maps and the layer EPSG: 4326 and WGS: 84.

## Results

### Identification and differentiation of *Brucella* isolates by *bcsp31*- and Bruce-ladder PCR

**Fig. 1:**
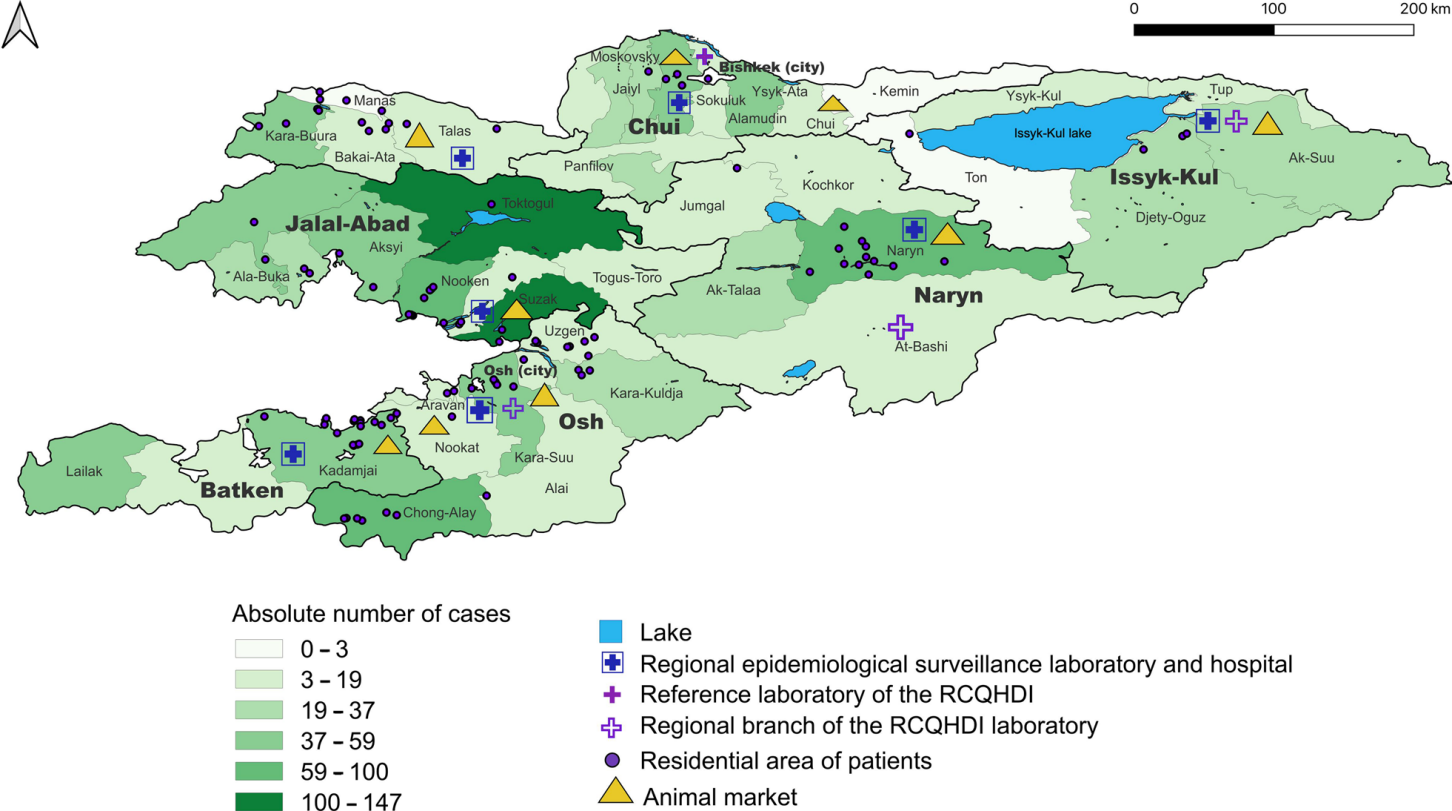
Residential areas of brucellosis patients December 2019 to November 2020. Prevalence of brucellosis by rayon (district) in 2019 and 2020 in Kyrgyzstan, based on data from the Republican Center for Quarantine and Highly Dangerous Infections Ministry of Health. The map was created using QGIS 3.22 Białowieza software which is available online https://qgis.org/en/site/forusers/download.html

In this study, 100 *Brucella* isolates have been isolated from different regions in Kyrgyzstan (Fig. 1, Additional file 1: Table S1, Additional file 5: Table S6). The age of the patients ranged between 5 and 70 years. Majority samples (67%) were isolated from age of 25 to 65 years. The 10%, 18%, 5% of the samples were isolated from children under 14, 15 to 25 and elderly of over 65 years, respectively. The majority of patients were male (*n* = 72) and 28 were female. Most of isolates were collected in the southern oblasts (regions), Naryn, Osh and Batken and especially in the bordering regions to Usbekistan. All isolates were identified as brucellae by *bcsp31*-PCR and were confirmed as *B. melitensis* by Bruce-ladder PCR.

### Seasonal and occupational distribution of human *B. melitensis* strains

**Fig. 2:**
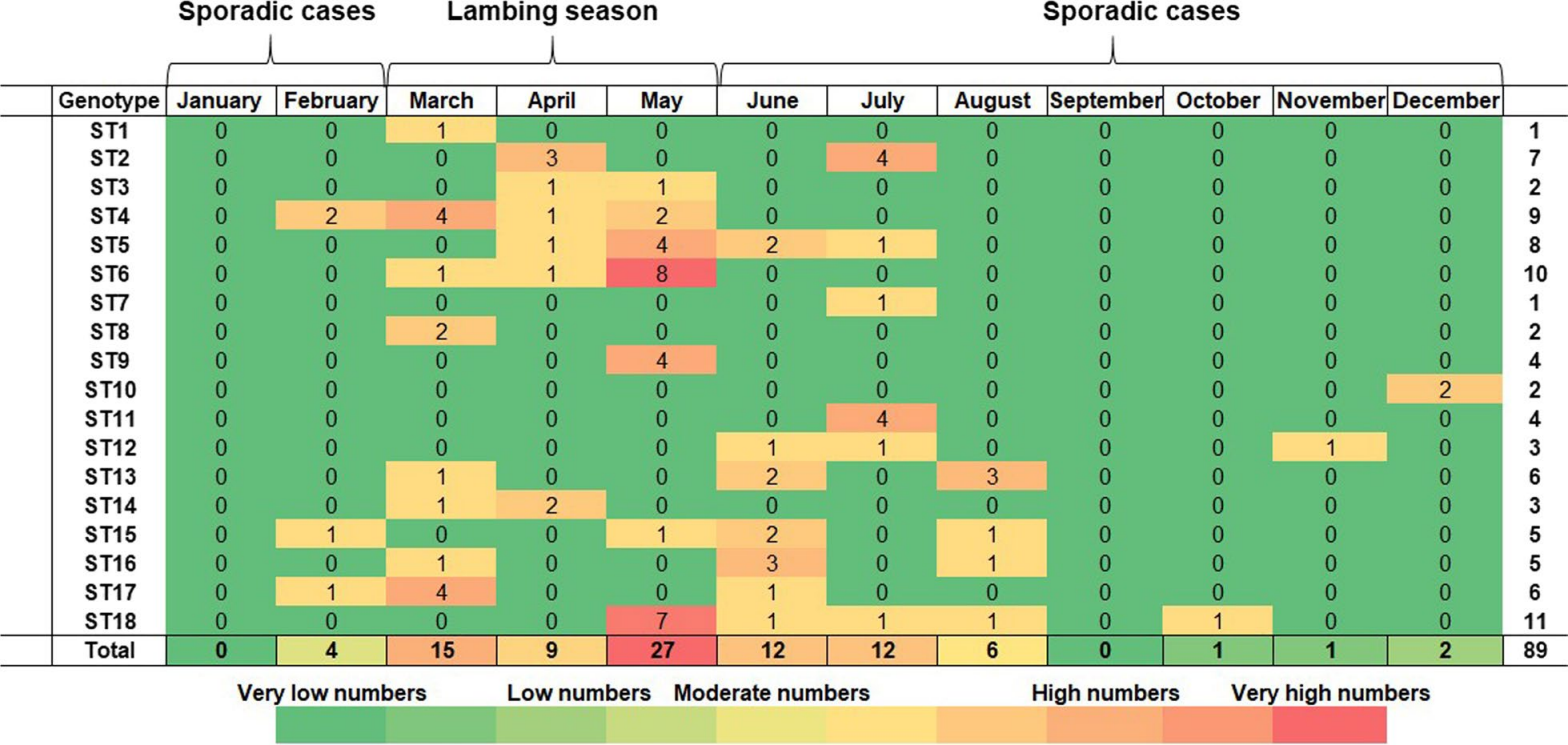
A heat map for isolation times of Kyrgyz *Brucella melitensis* strains isolates from December 2019 to November 2020. The isolated are assigned to single nucleotide polymorphisms (SNP) sequence types (ST), as given below

A heat map of the isolation times of *B. melitensis* strains by month shows that most isolates were collected from February to August in 2020 (Fig. 2). During the same period, the peak of the epidemic season was from March to July.

### Genome sequencing

Out of the 100 isolated strains, 89 isolates were sequenced (see Additional file 1: Table S1). Eleven isolates were not sequenced due to insufficient DNA. The average number of reads was 1,754,527.62 (min 504,098, max 4,080,054). The de novo assembled genomes comprised 22 contigs, on average, with an average coverage of 141.4-fold (min 41, max 327) and an average genome size of 3,290,169 bp (min 3,290,071 bp, max 3,290,266 bp) with N50 average values of 462,105 bp (see Additional file 3: Table S3). Sequencing raw data were submitted to the European Nucleotide Archive under the project number PRJEB53963.

### Core genome SNP typing of *B. melitensis*

**Fig. 3:**
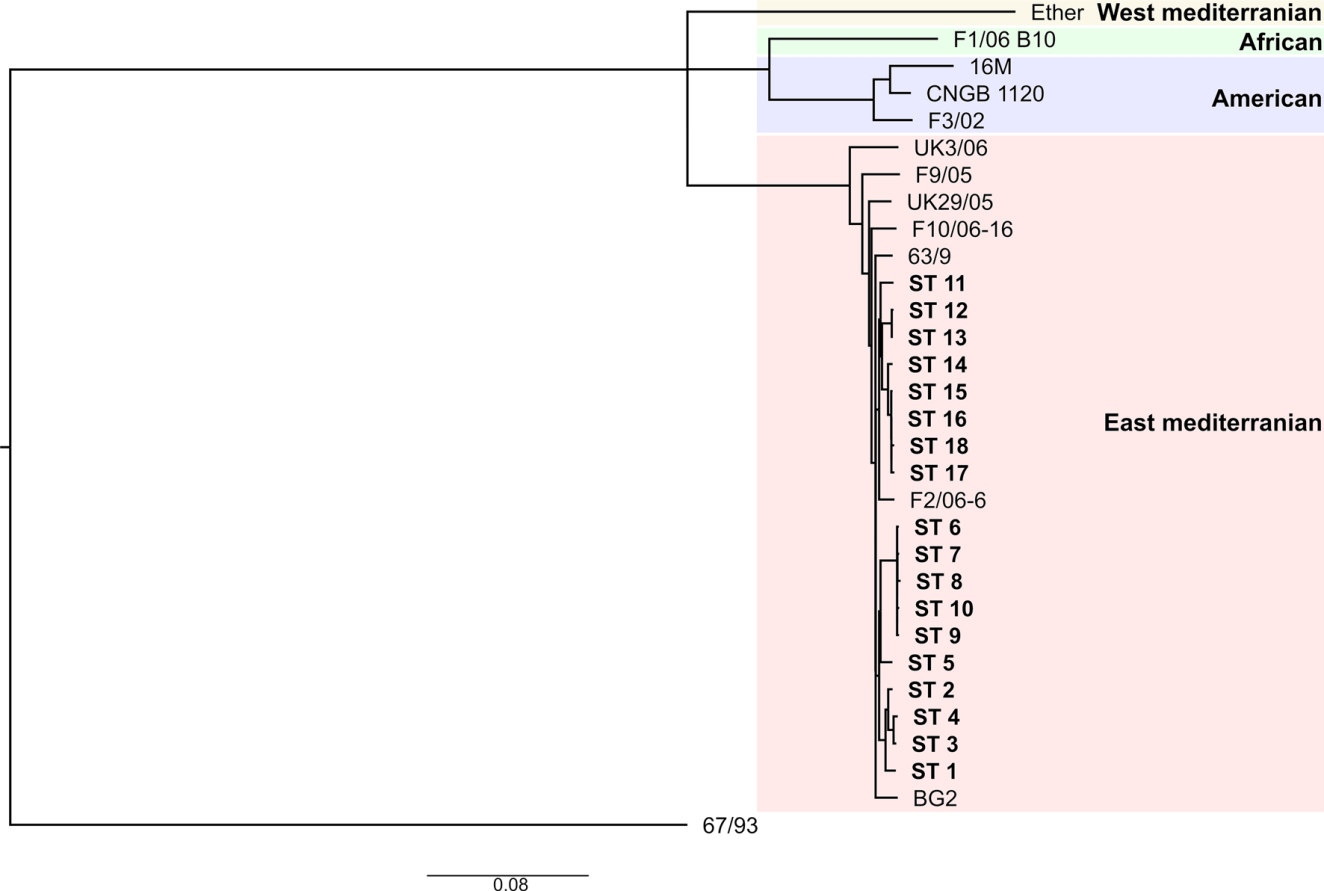
Maximum-likelihood tree based on core genome single nucleotide polymorphisms (cgSNPs) differences of the Kyrgyz *Brucella melitensis* strains to representatives of the known *Brucella melitensis* lineages. The bar indicates nucleotide substitutions per site

A SNP-based comparison of the Kyrgyz strains with 11 isolates representing the major genetic groups (Fig. 3) revealed that all of the Kyrgyz *B. melitensis* strains belonged to the East Mediterranean lineage.

**Fig. 4:**
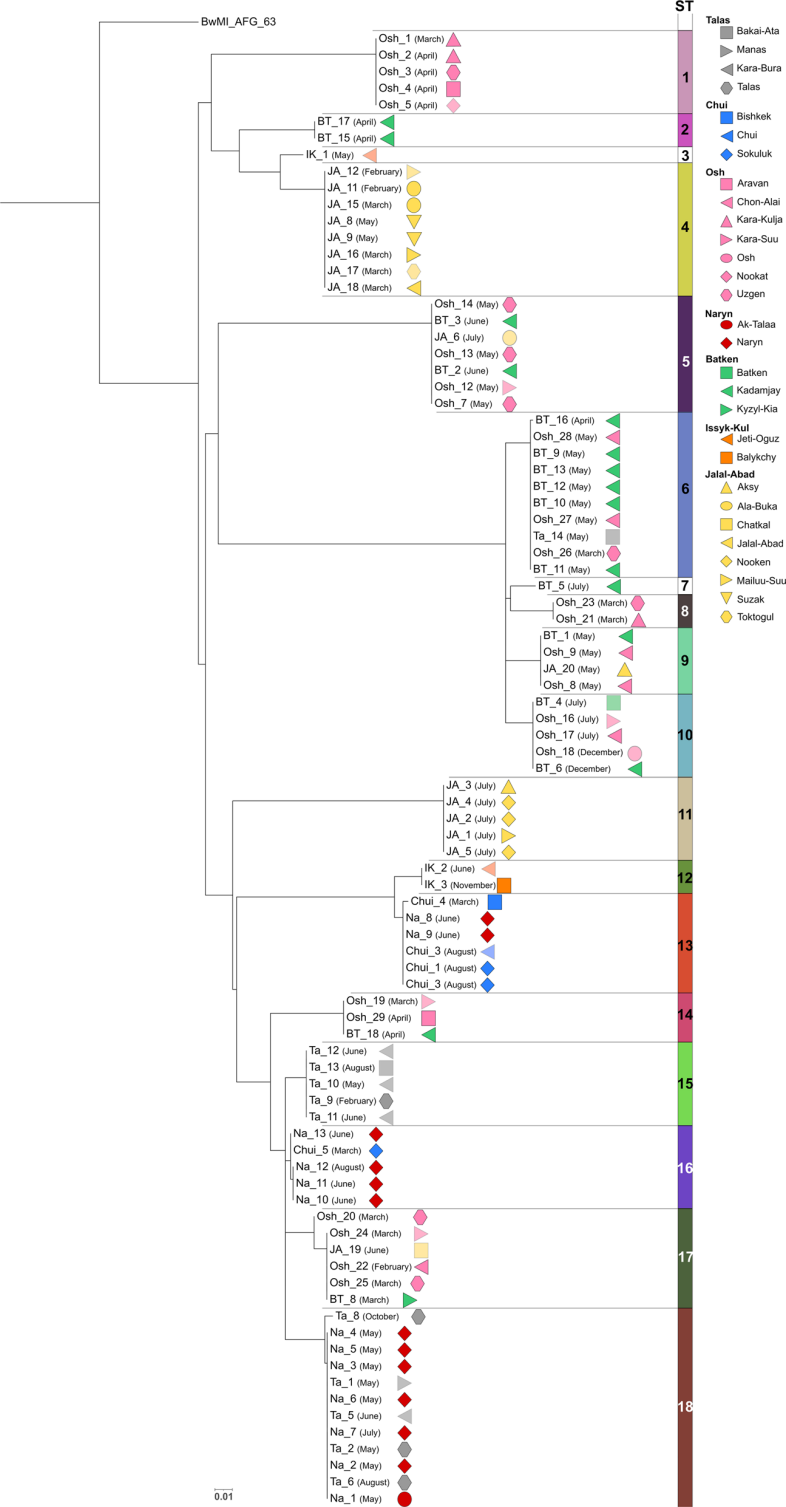
Maximum likelihood tree based on core genome single nucleotide polymorphisms (cgSNPs) of *Brucella melitensis* strains isolated in Kyrgyzstan. The columns with numbers indicate SNP sequence type affiliation. Symbols behind strain names give place of isolation. In brackets, the month of isolation is given. The bar indicates nucleotide substitutions per site

**Fig. 5:**
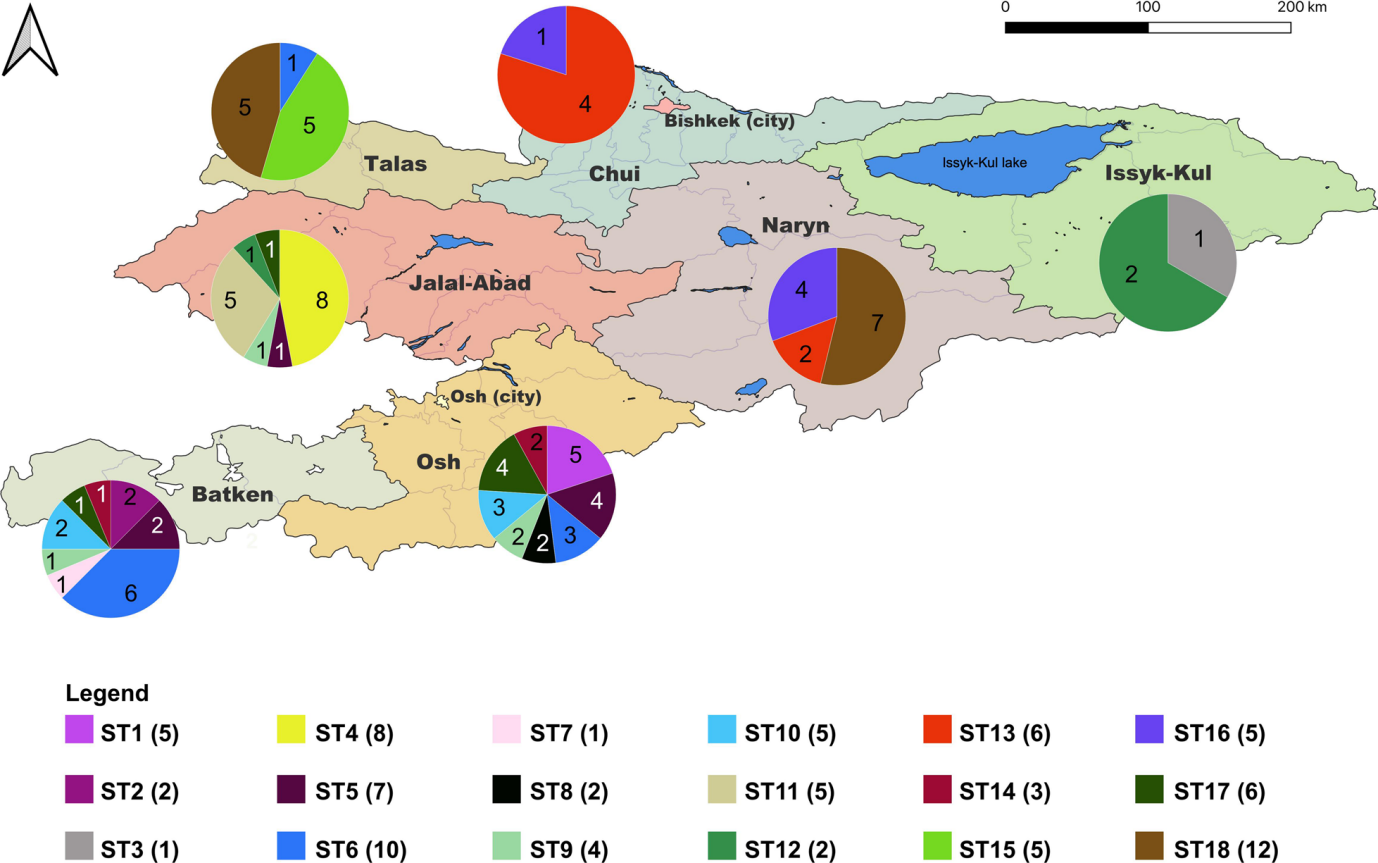
Genetic diversity of *Brucella melitensis* strains isolated in 2019 and 2020 from humans per oblast in Kyrgyzstan. Circles give number of isolates per sequence type isolated in the respective oblast. Numbers in brackets behind sequence types in the legend give sum of strains assigned to each sequence type. The map was created using QGIS 3.22 Białowieza software which is available online https://qgis.org/en/site/forusers/download.html

When comparing the Kyrgyz isolates in a core genome SNP analysis, 698 SNPs were identified, based on which 18 sequence types (ST) could be differentiated (Fig. 4). Strains of the same ST differed in at most five SNPs. Nine clusters comprised strains isolated in different oblasts, while seven STs originated from the same oblast and two were singletons. The latter, ST3 and ST7, were found in Issyk-Kul and Batken oblasts. The highest numbers of different sequence types were found in Batken (*n* = 8), Osh (*n* = 8) and Jalal-Abad (*n* = 6) oblasts (Fig. 5). *B. melitensis* strains collected from Batken and Osh oblasts were assigned to several ST.

ST18 represented the largest *B. melitensis* sequence type, with isolates collected in Naryn (*n* = 7) and Talas (*n* = 5) oblasts. Ten strains constituted the second most frequent sequence type, ST6, isolated from Batken (*n* = 6), Osh (*n* = 3) and Talas (*n* = 1) oblasts. Most strains of *B. melitensis* ST6 were isolated in May in the rayon Kadamjay (Batken). Four out of these six strains originated from the same village (Tamasha).

ST5, ST6, ST9, ST10, ST14 and ST17 each comprised between three and seven strains, which were mostly isolated from Osh, Batken and Jalal-Abad oblasts, all located in the East of Kyrgyzstan. The SNP comparison between ST5 and ST17 identified 120 SNP differences.

Seven sequence types (ST1, ST2, ST4, ST8, ST11, ST12, ST15) exclusively comprised strains from a single oblast (Osh, Batken, Jalal-Abad, Talas oblasts). For instance, ST4 and ST11 comprised eight and five strains from Jalal-Abad oblast, respectively. Despite their similar origin, the STs differed from each other by 133 SNPs. Further, it has to be noted that the period of isolation differed. While ST4 strains were mainly isolated in winter (February, March), the strains of ST11 were exclusively isolated in July. However, for other sequence types, there was no apparent association with the period of isolation.

ST5 and ST6 each comprised seven and ten *B. melitensis* strains, of which two strains each were found in members of the same family in Kadamjay rayon, Batken oblast. Moreover, ST9 comprised four *B. melitensis* strains, of which two strains were found in members of the same family in Chon-Alai rayon, Osh oblast.

The highest numbers of sequence types were found in May, June, and July with 27, 15, and 12, respectively. Notably, ST6 (*n* = 8) strains from Batken, Osh, and Talas oblasts and ST18 (*n* = 7) strains from Naryn and Talas oblasts were detected in May.

### Core genome SNP typing of *B. melitensis*

In silico MLST typing identified two known MLST sequence types (8 and 71), while for seven strains no sequence type could be assigned (see Additional file 4: Table S4). The latter all belonged to SNP sequence type 5. The majority of strains (*n* = 66) belonged to MLST sequence type 8 (SNP ST 6–18). The strains assigned to SNP ST 1–4 all belonged to MLST type 71. In contrast to the MLST analysis, in silico MLVA results were well in accordance with the SNP analysis-based clustering of the Kyrgyz strains. Allele profiles are available in Additional file 4: Table S5.

**Fig. 6:**
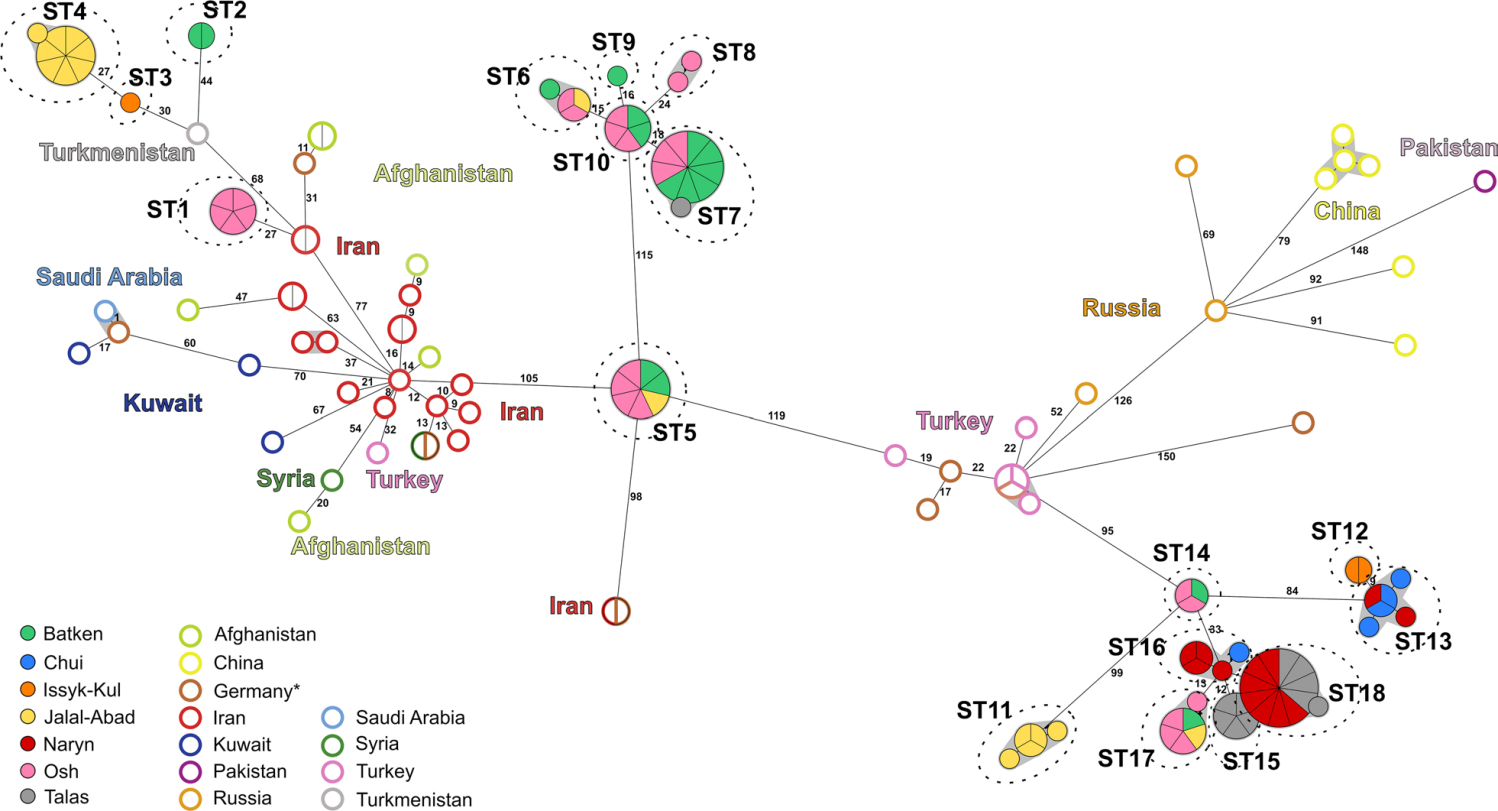
Minimum spanning tree based on allelic core genome multi loci sequence types (cgMLST) differences of Kyrgyz *Brucella melitensis* isolates and foreign strains. Dashed lines indicate identical sequence types (ST) identified based on nucleotide polymorphisms. Filled circles represent Kyrgyz strains with colors representing the oblast of isolation. Empty circles are foreign strains. Numbers on the lined indicate number of allelic differences. (*Probably imported cases)

A cgMLST analysis was performed to investigate the relationships between the 89 Kyrgyz *B. melitensis* strains and strains from Asian countries to place them in a (supra) regional context. A total of 54 strains from Turkey, Iran, Kuwait, Syria, Afghanistan, Turkmenistan, Saudi Arabia, Russia, Pakistan, and China were chosen for comparison (Fig. 6, Additional file 2: Table S2). Overall, the sequence types identified by cgSNP analysis also appeared as highly identical clusters in the cgMLST analysis, with at maximum 4 alleles difference between strains. Kyrgyz clusters were differentiated from each other by at least 9 alleles difference.

Further, the cgMLST analysis showed that Kyrgyz strains were most similar to strains from Turkey, Iran and Turkmenistan, although the allelic differences to foreign strains were comparably high. The closest matches were between ST3, which was a single isolate from Issyk-Kul, and an isolate from Turkmenistan (30 alleles difference) and strains of ST1, all isolated in Osh, strains from Iran (27 alleles difference). Only ST5 had an independent high similarity with the Iranian strains with a difference of 27 alleles. The Kyrgyz ST11–ST18 differed in at least 95 alleles from a Turkish strain.

## Discussion

This is the first study reporting the molecular epidemiology of brucellosis in humans in Kyrgyzstan. The objective of the study was to use molecular characterization of *Brucella* strains isolated from patients in all seven regions (oblast), in order to discover the species and genotypes circulating in Kyrgyzstan and their relationship to global genotypes.

Today, whole-genome sequencing is a widely employed molecular typing method for outbreak tracing and molecular epidemiology of brucellosis [26, 52]. In developing countries, such as Kyrgyzstan, genotyping of *Brucella* isolates is still not routinely performed, and thus it is often unknown which *Brucella* genotypes are circulating in humans and animal populations and tracking of outbreaks is hampered.

In Kyrgyzstan, according to the RCQHDI, a low incidence of human brucellosis was reported in the 1950–1990s but a high incidence in 1991–2011. In contrast, Kyrgyzstan, in the last decade, the numbers of human and animal cases of brucellosis have decreased significantly due to the use of vaccination in small ruminants [53]. However, human brucellosis is a continuing threat to Kyrgyzstan’s public health as it is still spread throughout the country’s farm animal population. According to the RCQHDI, a total of 1451 cases of human brucellosis has been reported and incidences of 13.4/100,000 (869 cases) and 8.8/100,000 (582 cases) in 2019 and 2020, respectively. During this period, the highest average incidence by oblast (regions) was estimated to be 47.6%, 34.9%, 22.1%, 16.6%, 15% in Naryn, Jalal-Abad, Talas, Batken, Chui and Issyk-Kul oblasts, respectively. During December 2019 to November 2020, most human brucellosis cases were reported from Toktogul, Suzak rayons in Jalal-Abad oblast, Chon-Alay rayon in Osh oblast, Naryn rayon in Naryn oblast and Kadamjay rayon in Batken oblast.

In the present study, 100 clinical isolates were collected from patients admitted to hospitals for treatment. All isolates were identified as *B. melitensis*. In humans, *B. melitensis* with its main reservoir in small ruminants, is the most important brucellosis agent followed by *B. abortus* (cattle) and *B. suis* (pigs) [54]. In this study, *B. melitensis* was the only species found and it proved to be prevalent in all oblasts. Kasymbekov et al. reported the isolation of 17 strains of *B. melitensis* from aborted fetuses of sheep (*n* = 15) and cattle (*n* = 2) in Kyrgyzstan which were the first *Brucella* isolates from Naryn oblast [55]. *B. melitensis* has been isolated from bovine milk, which was also demonstrated in a study conducted in the neighboring country Tajikistan [56]. This is in accordance with findings from other countries, where *B. melitensis* is regularly the predominate cause of human disease. Small ruminants serve as reservoir and source of infection for both, humans and other farms animal species, if cohabitation is practiced [57]. To prove the assumption that *B. melitensis* is indeed the main causative agent it is necessary to study the distribution of species and genetic relatedness of isolates from animal reservoirs and human patients. Our findings suggest that *B. melitensis* has spread all over Kyrgyzstan. However, molecular characterization of *Brucella* spp. in animal and wildlife is currently not conducted in the country.

Based on cgSNP analysis, 18 sequence types were identified. The majority of different sequence types were found in Batken, Osh and Jalal-Abad oblasts. These regions border each other and have contact via animal trade as well as use of the same pastures, fairs, or markets is usual practice. Also, the movement of people for social obligations i.e. cultural and religious gatherings will have an influence on the prevalence of certain sequence types if contaminated food is consumed during these events. Food might also be the vehicle for *B. melitensis* when it is sold countrywide or transboundary. Previous studies have shown that the consumption of cream/cheeses, which are prepared from raw milk, and not sufficiently heated grilled meat (shish-kebabs) is a risk factor in Leylek and Kadamjay rayons (districts), Batken and Jalal-Abad oblasts [58, 59].

Isolates from Kadamjay rayon (Batken oblast) and Alabuka and Nooken rayons (Jalal-Abad oblast) were isolated from areas close to the borders of Uzbekistan. We assume that the spread of bacteria in livestock between the two neighboring countries occurs during uncontrolled sharing of the same summer pastures. Transboundary trade is also usual practice [personal communication Abdyraev M. (principle adviser, scientist KRIV on 18.04.2022)]. We assume that Batken, Osh and Jalal-Abad oblasts seem to be the most likely sources i.e. reservoirs of infection for human brucellosis in Kyrgyzstan. Moreover, more than half of the human brucellosis cases of three oblasts were either due to very close transmission in one location or ongoing transmission between neighboring countries or oblasts. Three different sequence types were found in Naryn, Chui and Talas oblasts. This finding could be explained by the fact that livestock and dairy products from the Naryn oblast are mostly sold at markets or animal markets in Chui and other oblasts.

Currently, most rural residents owned animals. Based on isolates passport, four isolates were found in members of the same family in Batken oblast. Each family usually has four to seven persons. Nine patients had a history of handling cattle. Only for three strains from patients the source of infection was traced back to handling of small ruminants, the main host of *B. melitensis*. Most investigated isolates in this study came from patients in the age groups of 26–60 years (67%) and 15–24 years (18%), mainly from male adults (72%), which is in agreement with the fact that, worldwide, brucellosis is more common in men than in women [60]. In Kyrgyzstan, brucellosis is more prevalent in the young population, and males are more engaged in the care and management of farm and domestic animals and for this reason they may acquire the infection because of contact with infected animals [61].

According to the cgSNP analysis all Kyrgyz strains of *B. melitensis* belonged to the Eastern Mediterranean lineage. In addition, Kyrgyz *B. melitensis* strains were similar to strains from Iran, Turkmenistan and Turkey. Kasymbekov et al. proved that Kyrgyz *B. melitensis* strains from aborted fetuses of sheep and cattle isolated in Naryn oblast, seem to be genetically associated with the Eastern Mediterranean group of the *Brucella* global phylogeny [55]. These results hint at the transmission of *B. melitensis* between animals and human. Moreover, the countries of the Eastern Mediterranean share a long history of common trade as Kyrgyzstan was one of the most important geographical corridors of the old Silk Road between China, Central Asia and Europe and thus also a corridor for the spread of zoonotic diseases [62]. Liu et al. proved that *B. melitensis* strains from eleven countries along the Silk Road originated from the Eastern Mediterranean lineage and only one strain could be assigned to the Western Mediterranean lineage of *B. melitensis*. *B. melitensis* bv. 3 is the dominant biovar species and has been shown to be widely distributed [63, 64]. It also fits in this picture that *B. melitensis* strains isolated from humans in Kazakhstan were assigned to Eastern Mediterranean and Chinese origins [65–67]. This is in accordance with findings from China, a neighboring country to Kyrgysztan, where *B. melitensis* biovar 3 was found to be mainly responsible for human brucellosis between 2012 and 2016 in the province Guangxi. Moreover, all *B. melitensis* isolates in this study belonged to East Mediterranean lineage [68]. *B. melitensis* strains from China are genetically related to strains from other Asian regions such as Kazakhstan, Russia, Mongolia, and India [69]. The presence of epidemiologically related Eastern Mediterranean STs of *B. melitensis* in Kyrgyzstan was therefore not unexpected. For the moment, the entry and local spread of brucellosis in Central Asia remains unknown as there is limited information on the molecular epidemiology.

It can be assumed that the number of isolates collected in this study does not reflect the demographic situation and brucellosis prevalence in Kyrgyzstan. Isolates were only collected from patients who live close to regional hospitals, or in areas with good road connections to hospitals where medical care and treatment are easily accessible. The main transmission routes and the reasons for ongoing transmissions cannot therefore not be resolved in this study.

However, this study showed that different *B. melitensis* lineages are circulating in Kyrgyzstan and that there are trans-boundary transmissions between the oblasts and possibly also between countries. These results will provide the basis for further studies on the molecular epidemiology of *Brucella* circulating in humans and animals in Kyrgyzstan and the relationships of local isolates to those prevalent in neighboring countries. There is an urgent need to implement measures such as monitoring of animals for brucellosis, education of target groups, and strengthening of laboratory capacity in order to avoid further spread of brucellosis. Enforcement of movement control of animals within the country and strict restrictions on inter-regional movement are obvious needs to avoid spreading of animal diseases.

## Conclusion

The present study provides the first molecular study of *Brucella* in human in Kyrgyzstan. All isolates collected within 1 year were confirmed as *B. melitensis*, which usually mainly infects sheep and goats and is the most common species of *Brucella* in human illnesses. Our finding suggested that *B. melitensis* probably circulates among both small ruminants and cattle, because several patient isolates in this study confirmed that the source of infection was a cow. In addition, the distribution and regional clustering of the different sequence types allows conclusions to be drawn about epidemiological relationships between outbreaks. This study demonstrates the importance of sequencing-based monitoring of *Brucella* sp. for improving the prevention and control of human and animal brucellosis in the country, as strain identification and typing of *Brucella* isolates of animal origin has not yet been performed. That means genotyping of strains from human, livestock and wild animal species, as well as studies of seroprevalence in herds and risk factors on farms, are necessary for effective brucellosis control.

## Supplementary Information

**Additional file 1**. Metadata of Kyrgyz *Brucella melitensis* strains.

**Additional file 2**. Metadata of foreign *Brucella melitensis* strains.

**Additional file 3**. Genome assembly statistics.

**Additional file 4.**. Multilocus sequence typing (MLST) data and Multilocus variable number - tandem repeat analysis (MLVA) data.

**Additional file 5.** Prevalence of brucellosis in humans and number of sequenced strains (collected December 2019 - November 2020) in relation to number of populations by oblast in Kyrgyzstan. Based on data from the Republican Center for Quarantine and Highly Dangerous Infections of the Ministry of Health.

## Data Availability

The data presented in this study are openly available in the European Nucleotide Archive under the Project Number PRJEB53963 and in the additional file materials.

## Abbreviations

PCR: Polymerase chain reaction
RCQHDI: Republican Center for Quarantine and Highly Dangerous Infections Ministry of Health
SNP: Single nucleotide polymorphism
cgMLST: Core genome mulitlocus sequence typing
MLVA: Multiple locus variable number of tandem repeats analysis
DNA: Deoxyribonucleic acid
NBCI: National Center for Biotechnology Information
QGIS: Quantum Geographic Information System
ST: Sequence types
WOAH: World Organisation for Animal Health
